# Facial Neuralgia Treated by Nerve Vibration

**Published:** 1885-05

**Authors:** 


### Facial Neuralgia Treated by Nerve Vibration.

Facial neuralgia is such a painful disease that patients will submit to almost anything to obtain relief from their sufferings, and stretching nerves in the treatment of this affection has accordingly become quite an every day procedure, and in many cases has been fol.owed by satisfactory results. At the same time, one is naturally anxious to try every simple means of relieving pain, before advising one's patients to submit to such an operation; and the case reported by Dr. W. H. Neale (*Practitioner*, November, 1884), in which success was obtained by nerve vibration renders it worthy of being recorded. The case was that of a policeman, 35 years of age and of temperate habits, with no history of specific disease, and who attributed his neuralgia to exposure to rain For six years he had suffered almost constantly from severe facial neuralgia on the right side, and almost every drug in the pharmacopœia had been used without giving any permanent relief. All drugs were therefore abandoned and percussion was given a trial, a small, flat ivory disk, with about ninety vibrations to the second, being used, and applied at the most tender point in the right parietal region the pain was intensified at first but after about five minutes constant application the pain was gradually relieved, and in two or three minutes more was entirely absent. The disk was then moved along the forehead but no painful spot was found until it was placed under the right eye, just over the infra-orbital foramen. At this spot the patient complained of severe pain, and the right side of the face was drawn up for more than a minute. The muscles then gradually relaxed, and the pain soon disappeared. The disk was continuously applied until all pain had quite left. The patient was, after this, entirely free from pain for six hours, but he then thinks that he suffered as much if not more than usual. After this the percuteur was applied daily for two weeks to the various tender spots found out by running the percuteur lightly over the skin. When the spots were touched the patient immediately called out, and the application was continued until the pain died away. The improvement from the commencement of the treatment was almost constant; however, there were several severe relapses, during which the sufferings were even more severe than before the treatment was commenced. But after the applications had been continued for two weeks, the patient was entirely cured, and four months after this he reported as having been for all that time entirely free from pain.—*Therapeutic Gazette.*

